# Predictive nomogram for central lymph node metastasis in papillary thyroid microcarcinoma based on pathological and ultrasound features

**DOI:** 10.3389/fendo.2023.1108125

**Published:** 2023-07-06

**Authors:** Denghui Wang, Ji Hu, Chang Deng, Zhixin Yang, Jiang Zhu, Xinliang Su

**Affiliations:** ^1^ Department of Endocrine and Breast Surgery, The First Affiliated Hospital of Chongqing Medical University, Chongqing, China; ^2^ Department of General Surgery, The First People’s Hospital of Chongqing Liang Jiang New Area, Chongqing, China; ^3^ Department of Endocrine and Breast Surgery, The Central Hospital Affiliated Chongqing University of Technology, Chongqing, China; ^4^ Department of Breast and Thyroid, Guiyang City Maternal and Child Health Care & Guiyang City Children’s Hospital, Guiyang, China; ^5^ Department of Hepatobiliary Surgery, West China Hospital of Sichuan University, Chengdu, China

**Keywords:** nomogram, PTMC, CLNM, pathological, ultrasound

## Abstract

**Background:**

Central lymph node metastases (CLNM) in papillary thyroid microcarcinoma (PTMC) are common, but management through prophylactic central lymph node dissection (pCLND) remains controversial. In this study, the independent predictors of CLNM in PTMC were retrospectively studied based on ultrasound and pathological data, and we aim to establish the prediction model to predict CLNM in PTMC.

**Methods:**

This study included a total of 1,506 patients who underwent thyroid surgery for PTMC at the First Affiliated Hospital of Chongqing Medical University from 2015 to 2018. Ultrasound and clinicopathological features were summarized and analyzed. Univariate and multivariate analyses were performed to determine the risk factors associated with CLNM. The prediction model is established and verified according to the multivariate analysis results. The Kaplan–Meier curve was used to evaluate the effect of CLNM on survival.

**Results:**

The CLNM rate was 44.5% (670/1,506). Multivariate analysis showed that men, younger age, smaller diameter, ETE, microcalcification, without Hashimoto’s thyroiditis, and multifocal were independent risk predictors of CLNM. Nomogram has a good discriminative ability (C-index: 0.755 in the validation group), and the calibration effect is good. In the DCA curve, the CLNM prediction model performed better net benefit given any high-risk thresholds. The median follow-up time was 30 months (12–59 months), 116 cases were lost, and the follow-up rate was 92.8% (1,506/1,622). Of the 1,506 patients included, 12 (0.8%) experienced recurrence.

**Conclusion:**

The likelihood of CLNM can be objectively quantified before surgery by using this reliable and accurate nomogram that combines preoperative ultrasound with clinicopathological features. Clinicians can use this nomogram to assess central lymph node status in patients with PTMC and consider prophylactic CND in patients with high scores.

## Introduction

Presently, thyroid carcinoma incidence happens more frequently, making it the most ordinary malignant neoplasm of the endocrine mechanism all around the world. Specifically, papillary thyroid microcarcinoma (PTMC) is defined as a subtype of papillary thyroid carcinoma (PTC) with a maximal size of less than 1 cm, accounting for 30% of PTC cases (1, 2). The reasons for the increased incidence were always controversial, whereas it might be the extensive utilization of high-sensitivity diagnostic tools like ultrasonography that allows the detection concerning smaller nodules. This was despite the fact that PTMC was an indolent tumor with a 10-year disease-free survival rate of >91% and a 15-year disease-free survival rate of >87%. However, the presence of lymph node metastasis (LNM) was always associated with an unfavorable oncological outcome (3–5), with a 0.4% fatality rate (6). Many studies reported the highest risk of central compartment LNM (CLNM), between 18% and 80% (7, 8). CLNM was considered one risk factor for distant metastasis (9), with the potential for recurrence and concomitantly reduced survival rate (4, 9). There were, nevertheless, questions about the implementation of prophylactic central lymph node dissection (pCLND) for PTMC. It was suggested that pCLND might benefit patients with PTMC by reducing local recurrence and enhancing the disease-free survival rate (10–12). Others hold the opposing view that it did not improve survival but rather increased the risk of nerve injury and hypoparathyroidism (13–15). Although the American Thyroid Association (ATA) guidelines (version 2015) stated that thyroidectomy without pCLND was indicated for the noninvasive treatment of marginal (T1 or T2), cN0 PTC (16), the Expert Consensus on the Diagnosis and Treatment of Papillary Thyroid Microcarcinoma (version 2016) edited by Chinese academics, recommended the promotion of pCLND for PTMC in the context of this technical support (17). The rate of preoperative detection of CLNM was relatively low due to limitations in imaging technology and operators. For instance, the diagnostic sensitivity of CLNM in the USA was only 51%–58.3%, with a false-passive rate of 44.6% (18). An appropriate, noninvasive tool to quantify the risk of CLNM in this setting may be helpful in the management of patients with PTMC.

It is important to balance the harms and benefits of pCLND in patients with PTMC for individualized and precise treatment. It is reasonable not to perform pCLND in low-risk PTMC patients. This was because it would reduce the extent of the procedure and the associated perplexities, particularly with regard to the protection of peripheral nerves, and facilitate the patient’s postoperative recovery. In high-risk PTMC patients, the implementation of pCLND can achieve effective radical treatment of the tumor. Therefore, this paper not only identified risk factors for CLNM through intraoperative and preoperative findings but also established a predictive model for CLNM in patients with PTMC. This accurate and intuitive nomogram offered the possibility to objectively quantify CLNM preoperatively. At the same time, it achieved good utility and convenience in developing individualized treatment plans for patients.

## Materials and methods

### Patient information

The local institutional ethics committee backed up this retrospective research. Informed consent was waived after careful review by the ethics committee as there was no patient interest or privacy involved. The records of patients with PTMC who underwent surgery from January 2015 to December 2018 at the Department of Endocrinology and Breast of The First Affiliated Hospital of Chongqing Medical University were retrospectively reviewed. The total number of patients went through not less than a physical check, cervical ultrasound checking, and fine needle aspiration (FNA) before receiving surgery. Patients were the most vulnerable.

Patients were excluded from this research on the condition that they owned any of the elements below:

1. Non-PTMCs or mixed-type PTMC (14)
2. Reoperation or other head and neck surgery history (55)
3. Distant metastasis at diagnosis (7)
4. The radiation exposure during childhood or family PTMC history (3)
5. Incomplete clinicopathological data (144)
6. Those not to be follow-up after receiving surgery (116)
7. Adolescent (9)

**Figure 1:**
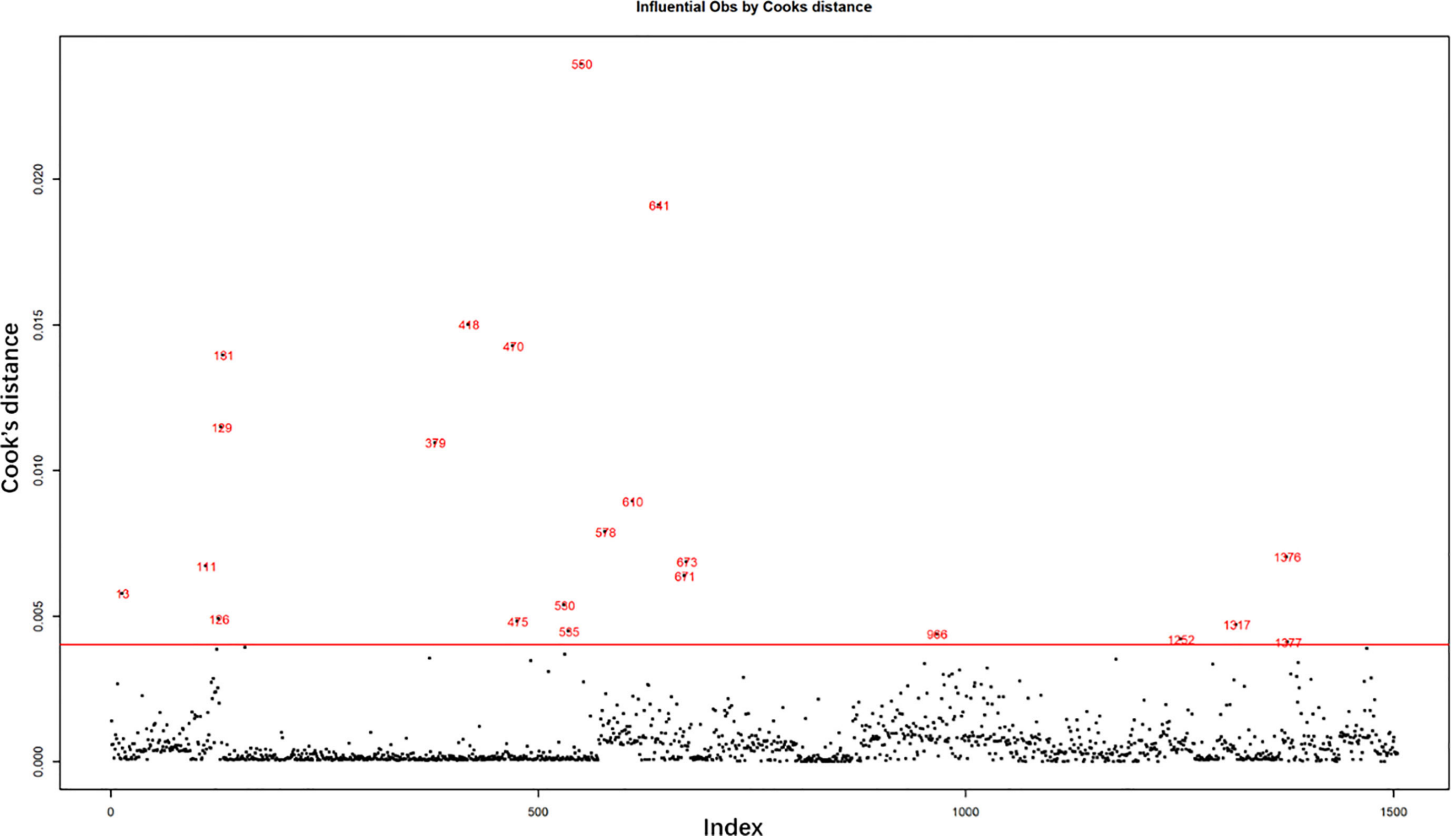
Excluded abnormal influential observations by Cook’s distance.

In total, 350 patients were excluded; additionally, 1,506 eligible patients were ultimately included in this research (**Figure 1**).

### Surgical procedures

After the patient finds a nodule of the thyroid through imaging inspection or palpation for the first time, we first perform the patient’s thyroid function test and the neck ultrasonography for diagnosis. Second, for nodules with suspected malignancy, the fine-needle aspiration (FNA) would be implemented. After all inspections are completed, we will communicate with the patient and their family members about the surgical method and surgical risks. The lobectomy plus ipsilateral central lymph node dissection (CLND) was the foundational therapy in a surgical manner. The total thyroidectomy plus bilateral multifocal CLND was deeply implemented for the cases suffering from bilateral multifocal tumors, tumors located in the thyroid isthmus with extrathyroidal extension (ETE), suspicious metastatic lymph nodes by preoperative examination or intraoperative frozen section biopsy showing CLNM. Lobectomy was stated as the removal of the implicated lobe, including the pyramidal lobe and the isthmus. Total thyroidectomy (TT) was stated as the removal of the two lobes: the pyramidal lobe and the isthmus lobe. Central lymph node dissection (CND) encompassed the removal of the pretracheal, prelaryngeal, and both the left and right paratracheal nodal basins (19). We routinely conducted a CLN intraoperative frozen section biopsy to further determine the degree of the lymph node analysis and the demand for total thyroid resection. Three pathologists independently and blindly diagnosed all resected lymph node and thyroid specimens, and a frozen section biopsy revealed CLNM.

### Clinicopathological and ultrasonographic data

We included the relevant data from ultrasonography and clinicopathological characteristics in the analysis. Data gathered included tumor size, age, multifocality, sex, ETE, preoperative cancer-related suspicion, central LN status, along with US features including shape, margin, location, echogenicity, length/width rate > 1, microcalcification emergence, the status of blood flow, and discontinuity of the capsule. Age has been dichotomized in view of the present stage criteria at 55 years. The biggest size of the neoplasm has been stated. The multifocalities have been characterized as > 1 overall neoplasm focus (either in the identical lobe or in the disparate lobes). In one single lobe, two or more PTMC foci were unilateral multifocality, whereas two or more PTMC foci in one lobe plus isthmus or both lobes were bilateral multifocality. Diagnosis of Hashimoto’s thyroiditis (HT) was made on the basis of the pathological data. ETE was based on pathological data and stated as the primary neoplasm spreading through the thyroid capsule to the perithyroidal soft tissue, including involving strap muscles or perithyroidal fat, or spreading to circumambient structures including trachea, larynx, recurrent laryngeal nerve, esophagus, skin, subcutaneous soft tissue, internal jugular vein, or carotid artery (20). The two radiologists, each of whom has over 15 years of expertise, implemented the overall preoperative US exams. Hypoechoicity, a length/width rate of less than one, fuzzy edges, hypervascularity, irregular forms, and the emergence of microcalcifications were the hallmarks of the malignant nodules. Pathologic lymph nodes (diffuse or focal, round form, cystic changes, internal calcification, and chaotic or peripheral vascularities on Doppler US) were stated as those with or without not less than one suspected US-type (focal or diffusion-like hyperechogenicity) (21). Ultrasound features of every nodule were encompassed as below: classification of the margin as to whether clear or irregular shape; whether the nodule was simply hypoechogenicity (inferior to the cervical band muscle); length/width ratio >1; of microcalcifications with a threshold value of 1 mm; discontinuity of the capsule defined as contact of the thyroid mass by means of the thyroid capsule, that is, >25% contacting with the neighbor capsule, is the best indicator for predicting the extrathyroidal spread (22); and rich and not rich blood flow status.

### Follow-up

For the total number of sick people, the initial follow-up was set at 1 month postoperatively; moreover, evaluation consisted of palpation and measurement of the levels of serum TSH. Serum thyroglobulin (Tg), along with Tg antibody levels, is merely for the patients who have undergone the overall thyroidectomy. Ultrasonography was performed 6 months after surgery. After that, the patient was re-examined every 6–12 months. On condition that the unstimulated Tg levels ≥ 2 ng/ml and stimulated Tg levels ≥ 20 ng/ml or serially elevated serum Tg (Tg-negative antibodies) are found, or if tumor recurrence is suspected on imaging studies (23), we will perform FNA in these patients, and if tumor recurrence is determined, the overall condition of the patient will be re-evaluated to decide on surgery method or other treatments.

### Statistical analysis

Categorical variables were summarized as numbers (percentage), and continuous variables were summarized as means (± standard deviation). One-way ANOVA test was conducted to test the correlation relationship of each variable with CLNM, and the *p*-value was displayed in the univariate analysis. A significance level of 0.05 was considered statistically significant for all statistical analyses. Prior to multivariate analysis, abnormal influential observations were examined using Cook’s distance, and the abnormal line was set as greater than six times the mean of Cook’s distance. Observations beyond the abnormal line were excluded from the multivariate analysis. The logistic regression model was built for the prediction of CLNM with all candidate factors as an entry in the independent variables, and then the variables were selected stepwise. The estimated coefficient of each selected variable and its corresponding 95% confidence interval were calculated, along with the *p*-value of each estimated coefficient. The model’s prediction discrimination was evaluated by C-index and ROC curve. AUC was exhibited with the ROC curve. The model’s calibration ability was evaluated by a calibration plot. The Kaplan–Meier curve was plotted for the overall survival grouped by CLNM, and the log-rank test was conducted accordingly. At last, decision curve analysis (DCA) was carried out to evaluate the net benefit of the prediction model with different risk thresholds used. In addition, the clinical impact curve was plotted along with the DCA curve to evaluate the discrepancy between the number of predicted positive and true-positive observations with different risk thresholds used.

## Result

### Baseline clinical and US characteristics of patients with 1,506 PTMC

The median follow-up time is 29 months. The baseline characteristics of risk factors for CLNM are shown in **Table 1**. Factors that exhibited significant association with CLNM *via* one-way ANOVA test were sex, length, invasion, preoperative clinical suspicion of CLNM, Hashimoto’s thyroiditis, laterality, focal infection, ETE, age, and diameter. Women accounted for 83.3% in the no-CLNM group and 65.2% in the CLNM group. Patients without preoperative clinical suspicion of CLNM accounted for 99.2% in the no-CLNM group and 83.7% in the CLNM group. Unilateral accounted for 93.9% in the no-CLNM group and 82.7% in the CLNM group. Unifocal accounted for 86.7% of the no-CLNM group and 61.2% of the CLNM group. Patients without ETE accounted for 97.6% of the no-CLNM group and 89.0% of the CLNM group. The average age in the no-CLNM group is 45.02 (SD = 12.3), and in the CLNM group, it is 41.46 (SD = 10.68). The average diameter in the no-CLNM group is 6.64 (SD = 2.07), and it is 7.03 (SD = 2.08) in the CLNM group. Apart from these factors, the rest of the factors do not show a strong discrepancy in terms of the distribution between the no-CLNM and CLNM groups.

**Table 1:** Univariate analysis of risk factors for central lymph node metastasis.

	*n* (%) or mean (SD)		*p*-value
	CLNM = No	CLNM = Yes	
Sex			
Male	140 (16.7%)	233 (34.8%)	<0.0001
Female	696 (83.3%)	437 (65.2%)
Age	45.02 (12.3)	41.46 (10.68)	<0.0001
Diameter	6.64 (2.07)	7.03 (2.07)	0.0003
Tumor location			
Upper	175 (20.9%)	132 (19.7%)	0.144
Middle	396 (47.4%)	310 (46.3%)
Lower	233 (27.9%)	188 (28.1%)
Isthmus	32 (3.8%)	39 (5.8%)
Margin			
Clear	228 (27.3%)	172 (25.7%)	0.395
Near-clear	330 (39.5%)	263 (39.3%)
Unclear	278 (33.3%)	235 (35.1%)
Shape			
Regular	193 (23.1%)	140 (20.9%)	0.23
Near-regular	359 (42.9%)	286 (42.7%)
Irregular or lobulated	284 (34.0%)	244 (36.4%)
Echogenicity			
Hypoechogenicity	773 (92.5%)	614 (91.6%)	0.614
Isoechoic	61 (7.3%)	55 (8.2%)
Hyperechoic	2 (0.2%)	1 (0.1%)
Length			
Width ratio ≤1	572 (68.4%)	490 (73.1%)	0.0462
Width ratio >1	264 (31.6%)	180 (26.9%)
Calcification			
No	347 (41.5%)	251 (37.5%)	0.663
Microcalcification	424 (50.7%)	385 (57.5%)
Coarse calcification	65 (7.8%)	34 (5.1%)
Blood signal			
No blood flow signals	419 (50.1%)	323 48.2%)	0.925
Enriched blood flow signal	68 (8.1%)	77 (11.5%)
Punctate flow signal	349 (41.7%)	270 (40.3%)
Invasion			
Continuity of the capsule	783 (93.7%)	609 (90.9%)	0.0439
Discontinuity of the capsule	53 (6.3%)	61 (9.1%)
Preoperative clinical suspicion of CLNM			
No	829 (99.2%)	560 (83.7%)	<0.0001
Yes	7 (0.8%)	109 (16.3%)
Hashimoto’s thyroiditis			
No	663 (79.3%)	570 (85.1%)	0.0039
Yes	173 (20.7%)	100 (14.9%)
Laterality			
Unilateral	785 (93.9%)	554 (82.7%)	<0.0001
Bilateral	51 (6.1%)	116 (17.3%)
Focal infection			
Unifocal	725 (86.7%)	410 (61.2%)	<0.0001
Multifocal	111 (13.3%)	260 (38.8%)
ETE			
No	816 (97.6%)	596 (89.0%)	<0.0001
Yes	20 (2.4%)	74 (11.0%)
Recurrence			
No	831 (99.4%)	663 (99.0%)	0.333
Yes	5 (0.6%)	7 (1.0%)
Month	30.74 (13.63)	30.11 (13.53)	0.372

The frequency of each category across CLNM is calculated for categorical variables along with its percentage; the mean and standard deviation are calculated for continuous variables; the ANOVA test of each variable versus CLNM is conducted to test their associations; and the p-value of the ANOVA test is recorded in the table.

### Result of Cook’s distance

The influential observations were examined by Cook’s distance, as shown in **Figure 1**. The horizontal red line is the abnormal line set for excluding abnormal influential observations beyond the line. It was set at six times the mean of Cook’s distance. A total of 22 abnormally influential observations were excluded, leaving 1,484 observations for the multivariate analysis.

### Comparison of clinical and US factors with CLNM in the training group

A multivariate analysis of the risk factors for CLNM was carried out using a logistic regression model. Data were randomly split by a ratio of 3:2 as training dataset and a validation dataset, respectively. All candidate variables were included at the beginning of the model selection and were selected stepwise. The selected variables are shown in **Table 2**, along with their odds ratio (OR), corresponding 95% CI, and *p*-value. Among the selected variables, the significant variables were sex, ETE, Hashimoto’s thyroiditis, focal infection, age, and diameter. Compared to men, women are less likely to experience CLNM, and their OR is 0.32 (95% CI: 0.22–0.45). Patients with ETE are much more likely to experience CLNM (OR = 10.72; 95% CI: 4.08–28.12). Patients with Hashimoto’s thyroiditis are less likely to experience CLNM (OR = 0.53; 95% CI: 0.36–0.80). Compared to unifocal infection, the odds of experiencing CLNM is 4.33 (95% CI: 3.04–6.17) times higher for multifocal infection. The age increases by 1 year, and the odds of CLNM would decrease by 0.03 (OR = 0.97; 95% CI: 0.96–0.98). If the diameter increases by 1 mm, the odds of CLNM would increase by 0.08 (OR = 1.08; 95% CI: 1.00–1.16). Patients with microcalcification are more likely to have CLNM (OR = 1.24; 95% CI: 0.91, 1.68), and coarse calcification is a protective factor for the occurrence of CLNM (OR = 0.73; 95% CI: 0.38-1.38).

**Table 2:** Multivariate analysis of risk factors for central lymph node metastasis using logistic regression.

	Odds ratio	95% CI	*p*-value
Sex			
Male	Reference		<0.0001
Female	0.32	(0.22, 0.45)	
Age	0.97	(0.96, 0.98)	<0.0001
Diameter	1.08	(1.00, 1.16)	0.0135
ETE			
No	Reference		<0.0001
Yes	10.72	(4.08, 28.12)	
Calcification			
No	Reference		
Microcalcification	1.24	(0.91, 1.68)	0.0178
Coarse calcification	0.73	(0.38, 1.38)	0.0331
Hashimoto’s thyroiditis			
No	Reference		0.0026
Yes	0.53	(0.36, 0.80)	
Focal infection			
Unifocal	Reference		<0.0001
Multifocal	4.33	(3.04, 6.17)	

The C-index of the logistic regression model is 0.755.

### Validation of the prediction nomogram

**Figure 2:**
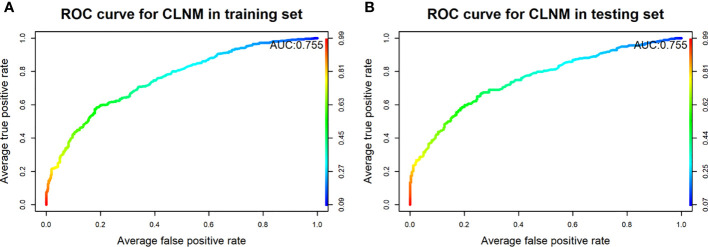
Receiver operating characteristic curves of the model for predicting CLNM in the training set **(A)** and testing set **(B)**.

**Figure 3:**
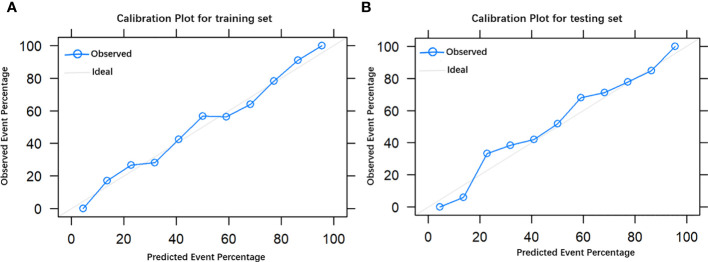
The calibration plot of the model in the training group **(A)** and testing group **(B)**.

**Figure 4:**
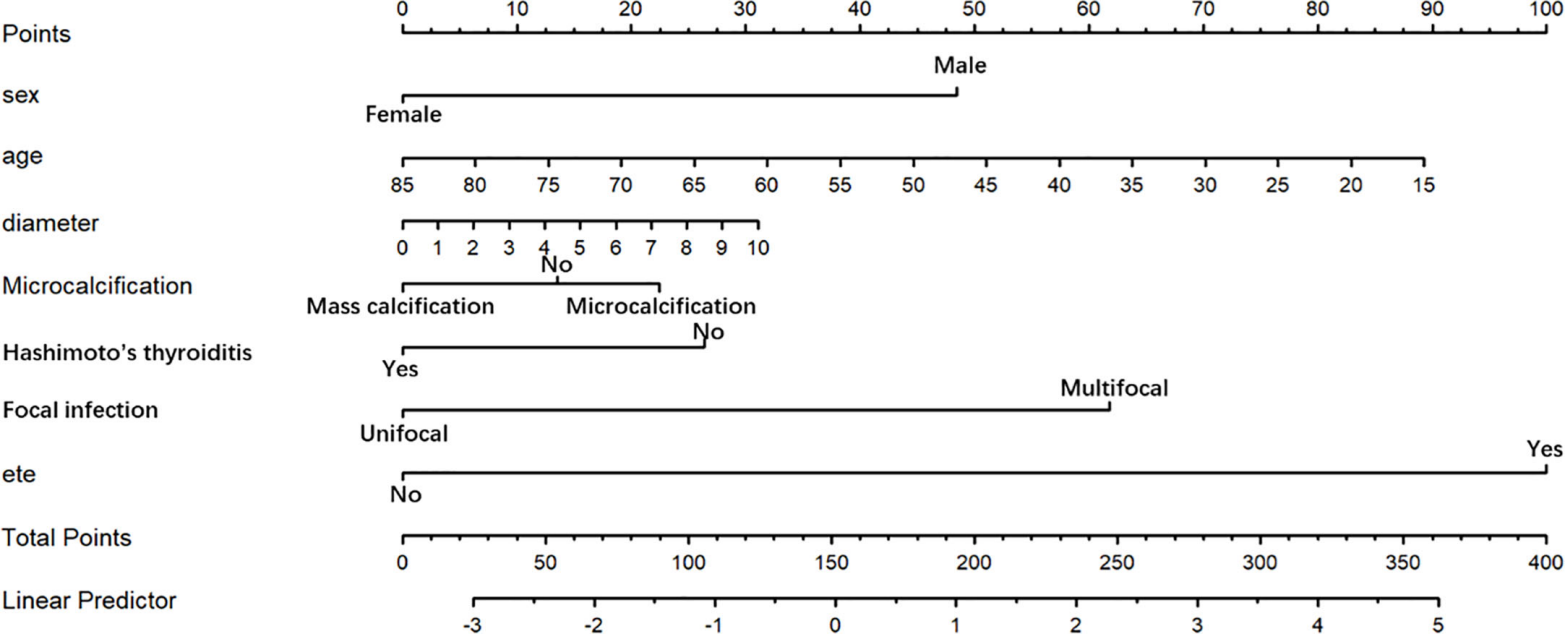
Nomogram for prediction of CLNM. A line is drawn straight up to the point axis that corresponds with each patient variable to obtain the points. The sum of these points is located on the total score point axis. A line is drawn downward to the risk axis to determine the possibility of CLNM.

**Figure 5:**
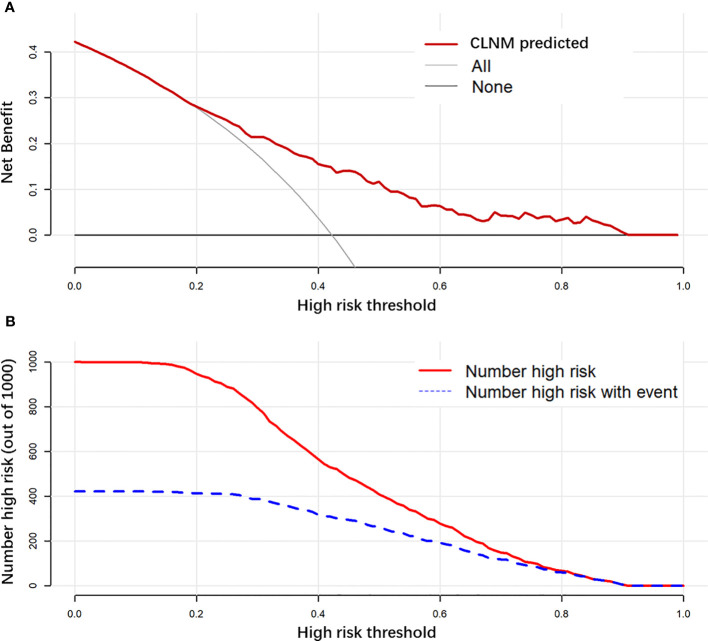
Decision curve analysis (DCA) is used to evaluate the net benefit of a prediction model with different risk thresholds used **(A)**. Clinical impact curve to evaluate the discrepancy between the number of predicted-positive and true-positive observations with different risk thresholds used **(B)**.

The final model achieved a C-index of 0.755 on the validation dataset. ROC curves for the training and validation sets are shown in **Figure 2**. The AUC on the training and validation sets was all 0.755, which indicates that the model can achieve good accuracy of prediction in both the training and validation sets. In addition, the calibration plots for training and validation are shown in **Figure 3**, and both calibrated lines are very close to the diagonal line, which indicates that the model was well calibrated. The predicted effect of each variable was visualized in the nomogram (**Figure 4**). The ETE has the most effective potential to explain the risk points in this study, followed by age, focal infection, sex, and diameter. Hashimoto’s thyroiditis and microcalcification had a modest contribution to the risk of CLNM. Finally, the model was further analyzed using DCA. The DCA curve and clinical impact curve were plotted (**Figure 5**). In the DCA curve (**Figure 5A**), the CLNM prediction model performed better in terms of net benefit given any high-risk thresholds. In the clinical impact curve (**Figure 5B**), the discrepancy of the number of CLNM between predicted positives and true positives converged drastically around 0.4. In order to have a better net benefit and a small discrepancy between the number of predicted positives and true positives, 0.4 would be an appropriate cutoff value for the prediction model in this study.

### Follow-up status

**Figure 6:**
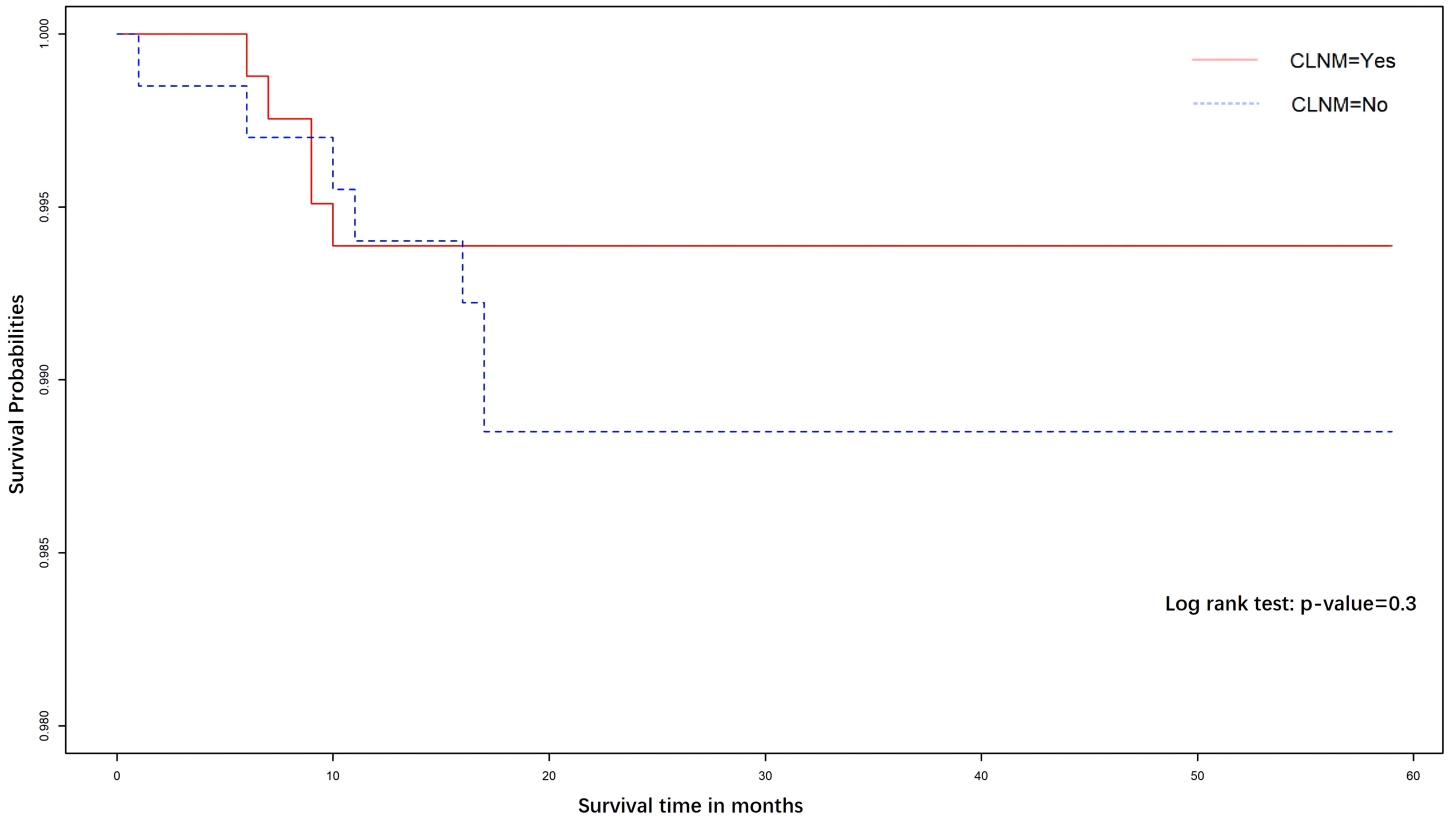
The Kaplan–Meier curves for the PTMC patients. There was no significant difference in the DFS rate between the CLNM(+) and CLNM(−) cohorts.

As last, the survival analysis of CLNM was conducted using the Kaplan–Meier curve. We followed up 1,506 patients (follow-up rate: 92.98%) after the initial surgery until December 2019. The interesting event is a recurrence of the PTMC. The *p*-value for the log-rank test is 0.3, which means that there is no significant difference between CLNM and no-CLNM group in terms of the recurrence rate. Both groups had a very low recurrence rate (**Figure 6**).

## Discussion

PTMC is a subtype of PTC that is less than 10 mm in diameter. In recent years, its incidence has gradually increased due to advances in ultrasonography and the importance individuals place on physical examination (24, 25). Despite the good prognosis of PTMC, a small number of patients developed local recurrence or distant metastases. Central LNM was only one of the essential factors contributing to their occurrence (26). Ultrasonography was not sensitive to detect metastatic central cervical lymph nodes prior to surgery. This was because they were usually marginal, confused by the overlying thyroid, or had atypical ultrasound features. However, many PTMC patients who were clinically lymph node-negative (cN0) were found to have CLNM metastases during surgery. CLNM (found only on pathological examination) was reported in 49.2%–64.1% of patients with cN0 PTMC (27, 28). In low-risk patients with papillary thyroid cancer, pCLND remained in doubt. Some studies advocated regular CLND during primary thyroidectomy in patients with PTMC. This was because it could benefit mPTC patients by reducing local recurrence and improving disease-specific survival rates (29, 30). It was also opposed because it did not improve survival but rather exacerbated the risk of neurological damage and hypoparathyroidism (14). Even in the research by Sugitani et al. and Ito et al., they found a lower incidence of tumor growth and new LNM in patients with low-risk (T1aN0M0) PTMC through prospective clinical studies. In patients who underwent surgery for tumor growth or new LNM, there were no life-threatening recurrences or deaths from thyroid cancer. There were also no distant metastasis or deaths from thyroid cancer in the active surveillance (AS) (31, 32). Therefore, accurate prediction of CLNM in PTMC is important for individualized treatment.

In this research, the incidence of CLNM was 44.5%, which was in line with the results of earlier studies (7, 8). As one of the important tools for the preoperative assessment of PTMC, ultrasound examination has been used as a routine for PTMC in China. However, the sensitivity and specificity of using ultrasound techniques to assess CLNM were 12.5% and 95.2%, respectively (33), due to a high dependence on the experience of the sonographer and interobserver variability (34). Therefore, the goal of this study was to develop a nomogram that could be used as an emerging strategy to personalize and quantify the likelihood of CLNM in patients with PTMC. The current research indicated that age, gender, tumor dimension, microcalcification, HT, focal infection, and ETE were independent predictors of CLNM. Tumor location, echogenicity, length/width ratio >1, blood signal, capsule invasion, and laterality were not predictive.

For a long time, age was for a clinically important prognostic factor in PTMC. Research by Ito found that younger age was only an independent indicator of PTMC progression (included: (1) increase in volume; (2) new manifestations of LNM; (3) clinical disease progression with an increase in tumor volume of 12 mm or greater; and new manifestations of LNM). In other studies, age had the same effect on PTMC (35, 36), that is, the lowest PTMC promotion rate in older patients and the highest in younger patients (3). In the present study, high age was a separate protective factor for CLNM. In addition, low age had the highest corresponding rank in the nomogram. In clinical practice, a more thorough preoperative assessment of the CLNM status of younger patients is necessary. Moreover, treatment may be different for younger patients than for older patients. Older patients with PTMC are the best candidates for observation, whereas younger patients may require active intervention.

Numerous studies showed that the incidence of PTMC was higher in women than in men, while the incidence of CLNM was low (37, 38). In this research, the ratio of men to women suffering from CLNM was approximately 1:2, and women were an independent protective factor for CLNM in patients with PTMC (OR = 0.32; *p* < 0.0001). It was traditionally assumed that the larger diameter of the tumor, the more aggressive it would be. ATA guidelines indicated that pCLND might be considered for patients with advanced primary neoplasms and clinically involved lateral cervical lymph nodes (cN1b). In contrast, in patients with marginal (T1 or T2), clinically negative PTC, and noninvasive patients (16), thyroidectomy may be appropriate instead of PCL. However, the incidence of CLNM in PTMC was usually about 50%, and there might be residual metastatic lymph nodes if pCLND was not performed, leading to resurgery and the risk of serious postoperative complications. For PTMC, some studies suggested that the risk of CLNM metastasis was higher if the diameter was larger (33, 38), and the same conclusion was reached in this study.

HT was the most common autoimmune thyroid disease, with an incidence rate of about 3.5 to five cases per 1,000 people per year (39, 40). The relationship between HT with PTMC has been controversial since it was first described by Dailey et al. in 1955 (41). A few scholars believed that HT was a protective factor for CLNM in PTMC (42, 43), while other studies showed no significant protective effect of HT on CLNM (44). In this study, HT was found to reduce the incidence of CLNM in PTMC. This might bring about fibrosis and atrophy of the thyroid due to HT-induced inflammation. This process involved an associated injury to the adjacent lymphatic vessels, disrupting PTC lymphatic diffusion, and eventually creating problems in reducing the likelihood of CLNM (45).

Bilateral or multifocal tumors might be caused by a single primary tumor spreading through the gland or by multiple concurrent primary tumors (46). It occurred in about 20%–40% of patients with PTMC (47). Although multifocal PTMCS were probably only synchronous tumors and not metastases from the largest primary neoplasm, they were associated with an increased risk of LNM, persistent local disease, distant metastasis, and local recurrence. In this study, it was analyzed in a single-bilateral lesion group and a single-multiple lesion group. The results found that multiple lesions were an independent risk factor for the development of CLNM in patients with PTMC (OR = 3.95; *p* < 0.0001) and the second significant variable in the nomogram.

Ultrasound was a convenient and quick way to evaluate PTMC. It might contain low central lymph nodes (grade VI) due to the presence of the thyroid or some anatomical areas (such as deep bony or air-filled structures and in the retropharyngeal region and mediastinum) that were not clearly visible on ultrasound (48). Retrospective research reported that the sensitivity of high-resolution ultrasound in predicting central LNM in PTC was only 38%. Seven characteristic variables of ultrasound were included in the analysis of this study. Microcalcification was one of the highly specific signs in ATA 2015 (16) and an essential ultrasound finding for suspected malignant nodes. It presented as punctate hyperechoic and was not easily missed. Nodules with microcalcification might be more likely to have cervical LNM. However, as hyperechoic indicated calcification, the ultrasound showed oxalates and concentrated colloids, which made it more difficult for junior doctors to identify microcalcifications. The ultrasound examinations enrolled in this study were performed by experienced sonographers, which marginally improved the diagnostic accuracy of microcalcifications. In this study, microcalcifications had an OR of 1.24 and were an independent risk factor for the occurrence of CLNM, which was consistent with other studies (32, 49). Microcalcifications reflected the rapid proliferation of cancer cells and the deposition of calcium salts due to the enlargement of blood vessels and fibers. As a result, if microcalcifications were found in the nodules, the status of LNs in the intermediate regions would need to be inspected more closely. Meanwhile, coarse calcification was found to be a protective factor for CLNM. This was probably because it was formed by necrosis within the tumor, indicating weak tumor growth activity.

ETE was a tumor that breached the thyroid capsule. After the thyroid capsule ruptured, the tumor was more likely to invade lymph nodes through the lymphatic vessels on the capsule surface. It involved the pharynx, strap muscles (thyroid gland of the sternum, thyrohyoid or mastoid), thyroid-bonding, larynx, prevertebral fascia, esophagus, mediastine, and even carotid artery, along with the PTMC artery. Various studies linked ETE to the invasiveness of PTMC. PTMC occurred when extrathyroidal structures were attacked and was shown to be a risk factor for CLNM (50, 51). According to the 2015 ATA criteria (16), ETE was only one indicator of a poor prognosis. In multivariate analysis, ETE was an independent risk factor for CLNM (OR = 10.72). In addition, ETE has the most effective potential to explain the risk points in the nomogram. This might be due to the fact that once the tumor cells breached the thyroid capsule, they metastasized to the CLNs through the surrounding abundant lymphoid tissue.

The results of the follow-up indicated that few patients suffered from central lymph node recurrence, which confirmed the reliability of examining the pathological status of the central lymph nodes. From the survival analysis, it was concluded that there was no significant difference in recurrence between patients with and without CLNM. However, some subclinical central lymph node metastases may lead to recurrence and distant metastasis. In addition, there is still controversy over whether PTMC patients need to undergo pCLND under domestic and foreign guidelines. Therefore, in order to achieve more accurate and individualized treatment, the model of this study was established to guide clinicians in selecting central lymph node dissection according to the scores, which were calculated based on disease status, so as to avoid unnecessary CLND and reduce postoperative complications. Moreover, in order to avoid recurrence, technical difficulties, hypoparathyroidism, and other complications caused by reoperation, CLND is still recommended when CLNM is found before or during surgery.

This research still had some shortcomings: (1) It was only a single-center retrospective research. Additionally, selection bias was inevitable. (2) Deficiencies in external validation. Relevant prospective multicenter clinical research should be implemented in the coming years to assess the validity of the proposed model. (3) The relatively short follow-up period in this study did not allow for the analysis of potential confounding factors for future metastasis. Therefore, more data and longer follow-ups are needed in the subsequent work. On the other hand, improving existing proven models, such as by including new variables that have been shown to work (such as BRAF-V600E), can improve the accuracy of the “old” nomogram. In summary, a quantitative CLNM prediction model for PTMC was formulated in this paper. Physicians can use the nomogram in this model to assess patients with PTMC in clinical practice based on clinicopathological features. For PTMC with a high possibility of CLNM, unnecessary pCLND may be avoided.

Above all, our study found that CLNM was independently associated with sex, age, diameter, ETE, calcification in ultrasound, Hashimoto’s thyroiditis, and focal infection. Using the above variables, we construct a nomogram. Clinicians can use the nomogram to assess central lymph node status in patients with PTMC. For patients with high scores, pCLND and meticulous postoperative evaluation can be considered.

## Data availability statement

The original contributions presented in the study are included in the article/Supplementary Material. Further inquiries can be directed to the corresponding author.

## Ethics statement

The studies involving human participants were reviewed and approved by Ethics Committee of the First Affiliated Hospital of Chongqing Medical University. Written informed consent to participate was waivered by the ethics committee.

## Author contributions

DW and JH: designed this study, analyzed the data, and wrote this paper. DW, JZ, CD, and ZY: data collection. XS: designed this study, conducted the examination, and modified the manuscript. All authors contributed to the article and approved the submitted version.
